# Does obesity rule out happiness? Preschool children’s perceptions of beauty-related happiness

**DOI:** 10.1186/s12887-022-03396-x

**Published:** 2022-06-11

**Authors:** Małgorzata Lipowska, Mariusz Lipowski, Natasza Kosakowska-Berezecka, Dorota Dykalska, Ariadna Łada-Maśko, Bernadetta Izydorczyk

**Affiliations:** 1 Institute of Psychology, University of Gdańsk, Bażyńskiego 4, 80-309 Gdańsk, Poland; 2 Department of Psychology, Gdańsk University of Physical Education and Sport, Gdańsk, Poland; 3 Institute of Psychology, Jagiellonian University, Krakow, Poland

**Keywords:** Body mass, Children, Physical attractiveness, Obesity, Gender stereotypes

## Abstract

**Background:**

Obesity is considered to be one of the most important factors reducing the sense of happiness and satisfaction with life, especially among women. This belief already exists in middle childhood, as the preschool period is a crucial point in the development of attitudes towards beauty. Preschoolers can identify physically attractive individuals, and they might already form attributions regarding the looks of adults (especially women), which in turn may constitute a foundation for their future concept of beauty-related happiness. Children’s attitudes towards the body are also strongly influenced by the content of gender stereotypes that prescribe and proscribe what women and men should look like. In our study, we aimed to analyse the relationship between associations of obesity and happiness made by preschool girls and boys (5-year-olds).

**Methods:**

A total of 680 families with five-year-old children (329 girls, 351 boys; *M*_*age*_ = 5.7 years) and both parents took part in the study. Children’s associations of different types of body sizes with perceptions of happiness were measured with the *Beauty & Health* pictorial scale.

**Results:**

Our results indicate that obese bodies were seen as unattractive, independent of gender (*p* < .001). Children associated looks with happiness—the body type identified as the most physically attractive was also seen as a happiest person. Lowest happiness scores were also ascribed to obese body types, but girls assessed men with a normal body type as happier than boys (*t* = 2.87, *p* = .004).

**Conclusions:**

Female bodies are already perceived along gender stereotypical lines at the age of 5, and are also related to potential predictions concerning women’s happiness. Children assessed female individuals with slim bodies, as well as those with normal weight, as happier than obese females.

## Background

Obesity is defined as a chronic disease characterized by the ratio between one’s body mass and height squared being 30 or greater [1]. Being obese has many negative consequences for a person’s physical [2–4] and mental health [5–7] and is related to disadvantageous economic outcomes [8]; hence, it naturally relates to various aspects of one’s life, including quality of life [9]. The concept of happiness is often defined as an overall measure of life evaluation, where happiness is defined as the degree to which a person positively evaluates the overall quality of their present life [10]. Multiple studies from many countries found evidence for a negative relationship between obesity and happiness (or life satisfaction) and a positive relationship between obesity and depression [11–16], but this relationship has rarely been analysed among preschool children and how they perceive the relationship between body size and happiness [17].

### Happiness according to children

Happiness, in general, is understood as manifestation of subjective well-being (SWB). The well-being of children is a multifaceted concept that refers to both their subjective feelings and experiences as well as to their living conditions [18]. From a child’s point of view, effective ways to experience happiness include having friendships, engaging in free play, experiencing nature, colours, or artwork, being challenged, and experiencing new things [19]. Children have their own specific agenda for happiness, which has been empirically identified. Research by Zawadzka and Dykalska-Bieck [20] has shown that the attributes of happiness in preschool children can be classified into three categories: family and friends, possessions, and positive emotions. Most preschoolers (71%) consider themselves to be happy; 12% of children consider themselves to be happy only sometimes, and 15% believe that they are not happy [21]. However, one of the responses given by preschool children when asked about happiness is that [happy people are] successful people (‘‘they succeed in everything’’, ‘‘they do a lot of useful things’’) [21]. Parents usually trigger the development of certain concepts in their children, and despite the almost universal interest in the issue of happiness at different stages of human life, there is a gap in the literature examining how various parental attitudes and beliefs may regulate preschool children’s perceptions of happiness [20]. In our study, we focused on children’s perceptions of beauty-based happiness.

### Body size, happiness, and gender stereotypes

The concept of beauty ideals is developed during the socialization process, and one of its aspects concerns body size—which body size is considered ideal? The answer to this question can be found within contemporary content of gender stereotypes that include prescriptions and proscriptions concerning women’s and men’s ideal looks [22–25]. As a result, both cultural norms transmitted by parents/caregivers and peers indirectly and directly determine children’s relationships with physical attractiveness, and influence their roles in their child’s social life [26–28].

One of the negative manifestations of such a relationship is body stigmatization [29, 30], which is already present in childhood [31, 32]. Children already prefer slim bodies and average-sized individuals by the time they reach preschool age [33, 34], and they already manifest a disinclination towards obese individuals by the age of three [35]. Three-year-olds ascribe negative features to obese individuals and are more likely to choose children of average build rather than overweight children as playmates [36].

Three- and four-year-old children are less likely to ascribe positive adjectives to individuals of average build than five-year-old children; this can be considered through the lens of the work of Inhelder and Piaget [37], according to which younger children rely mainly on dichotomous comparisons. Only at the age of five years do children begin to understand relationships based on seriation. Thus, it is difficult to state whether their preference for slim bodies is a result of body stigmatization or simply because the category “slim-good” appears earlier in development than “fat-bad”. In the research by Harriger [38], three-year-olds selected obese children as playmates more frequently than five-year-olds did. This is an interesting result, especially given that the three-year-olds also exhibited a significant inclination towards ascribing positive features to slim individuals and a general disinclination towards the bodies of obese individuals as well as those of average build; this suggests that the belief that “slim is better” increasingly appears in younger children. The results of these studies suggest that there is a difference between knowledge regarding stereotypes and stereotypical beliefs: children may exhibit knowledge of stereotypes independently of whether they believe the stereotypes are appropriate [38].

Stereotypical ways of thinking about being overweight or obese are reinforced between the ages of five and eight years [39]. Obese individuals are often discriminated against because their condition is associated with weak character rather than medical problems. Many perceive obesity as a sign of laziness, incompetence, lack of discipline, indulgence of one’s whims, and emotional problems [40].

Obese children are socially rejected by their peers [41] and, worse, by adults [42, 43]. Even young children believe that obese individuals are characterized by negative personality features and are worse behaved (e.g., more aggressive) and hence that they are inferior candidates for friendship [44]. Children also display a tendency to attribute reduced intellectual skills (slimmer is smarter) as well as interpersonal attractiveness (they are better partners to play with) [45] to such people, which can lead to the creation of specific types of sociometric statuses depending on body type.

A very interesting pattern is observed in the preschool period; this stage constitutes a crucial point in the development of attitudes towards both the beauty of the human body and gender stereotypes: preschoolers are able to identify physically attractive individuals, but do not compare their own self-image to this pattern [46]. For example, four-year-old girls express some anxiety related to their looks, but this does not influence their overall level of happiness [47]. Furthermore, preschoolers do not associate the looks of boys and girls with life success and happiness (no attributions of “slimmer is happier”, and “obese is the most miserable” occurred) [45], but they might form such attributions regarding the looks of adults. In our study, we aimed to analyse the relationship between associations of beauty and happiness made by preschool pupils (5-year-olds).

### Study overview and hypotheses

In our study, we aimed to analyse the relationship between associations of obesity and happiness made by preschool girls and boys (5-year-olds). We predicted that children would show a tendency to perceive slimmer silhouettes as more beautiful/handsome than obese silhouettes (H1) and that children would associate happiness more strongly with slim silhouettes than with obese silhouettes (H2). This would be moderated by a person’s gender—namely, this relationship would be stronger for female silhouettes than male silhouettes, indicating that, by the age of five years, children already associate happiness with one’s body shape/type; this would mainly be observed with regard to female bodies (H3).

Our study also allowed us to explore other relationships between perceptions of different categories of happiness and beauty separately for boys and girls. Many studies conducted among adults have shown a negative relationship between body size and happiness (or life satisfaction) [11, 12], but to our knowledge, this relationship has not been analysed among preschool children. Analysing such associations and their strengths and development among children might be an important line of research for future interventions aimed at improving the quality of life of teenagers and adults.

## Materials and methods

### Participants

A total of 680 families with five-year-old children (329 girls and 351 boys; *M*_*age*_ = 5.7 years, *SD* = 0.32) took part in the study. The age of the children was uniform to exclude its influence on the factors assessed in the study and because stereotypical perceptions of overweight manifest between 5 and 8 years of age [39]. It is worth noting that the term "preschool" has a different scope depending on the country, e.g., in Poland, it applies to children aged 2/3 to 5/6 years who have not yet started formal primary school education [48], even if participation in classes is compulsory (in Poland, the “zero” grade), but in the USA, the first year of formal education for children aged four and five years is called “kindergarten”. To control for the influence of familial factors and socioeconomic status (SES) on the children’s perceptions of happiness, data for several variables were collected: the age of the parents (mean age (*SD*) of the mothers was 33.62 (5.22) years; mean age (*SD*) of the fathers was 35.84 (5.52) years); the participants’ area of residence (28% lived in villages, 16% in small towns, 28% in towns, and 26% in big cities); and the number of children in the family (35% of the children were only children).

### Procedure

Data were collected from participants selected from preschools in the Pomeranian region of Poland. We randomly selected 20% of the educational establishments that run compulsory preschool education units (the so-called “zero grade”) and invited them to participate in the project. The data used for this study were part of a larger project, and the detailed recruitment procedure is described elsewhere [43]. Children were assessed individually by trained researchers (a psychologist, a PhD student, or a graduate student involved in the project) at educational centres. The work described was carried out in accordance with the Code of Ethics of the World Medical Association (Declaration of Helsinki) for experiments involving humans and using data collection. The protocol of this study was approved by the Ethics Board for Research Projects at the Institute of Psychology, University of Gdansk, Poland (decision no. 17/2013). The preparation of this paper was supported by grant 2015/17/B/HS6/04144 from the National Science Centre, Poland.

### Methods

The *Beauty & Health* (B&H) scale [49] is an illustrated questionnaire that allows the identification of how children attribute social success (happiness) and health-related behaviours to male and female silhouettes with distinctly different body weights. The scale comprises 18 questions. The B&H scale contains two subscales: Subscale 1 addresses pro- and anti-health behaviours (Healthy Behaviour, Cronbach’s α = 0.86), and Subscale 2 addresses assessing a person’s happiness (Social Success, Cronbach’s α = 0.89). In this study, we only used the Social Success subscale because its items depict children’s associations with Happiness [49].

When conducting the test, the children were shown a chart with three types of adult male and female silhouettes: slim, normal, and overweight. The children were asked to point to the silhouette that best fit the descriptions given by the researcher. The children were asked the following 6 questions/tasks from the beauty-related happiness subscale of the B&H scale: 1) Which woman/man is the prettiest/most handsome? (attractiveness of the silhouette); 2) Which woman/man has the most friends (number of friends); 3) Which woman/man earns the most money (being rich); 4) Which woman/man is the smartest (smartness); 5) Which woman/man is the nicest (niceness); and 6) Which woman/man works in the coolest place (having a good career). Each question could be analysed separately, but this method also allowed us to show the children’s overall perceived levels of happiness of the 3 female and 3 male silhouettes that were shown. A picture scored a point each time it was chosen by a child, so it was given 0 points if it was never chosen and 6 points if it was chosen in response to all six questions. The *Beauty & Health* scale has been validated in the population of children aged 5–9 years [49].

### Statistical analysis

Statistical analyses were performed using Statistica 13.1 for Windows (licenced to University of Gdańsk). A Pearson chi-square test and Friedman’s ANOVA was carried out to show significant differences between distributions.

## Results

### Assessment of the attractiveness of various body types

**Fig. 1:**
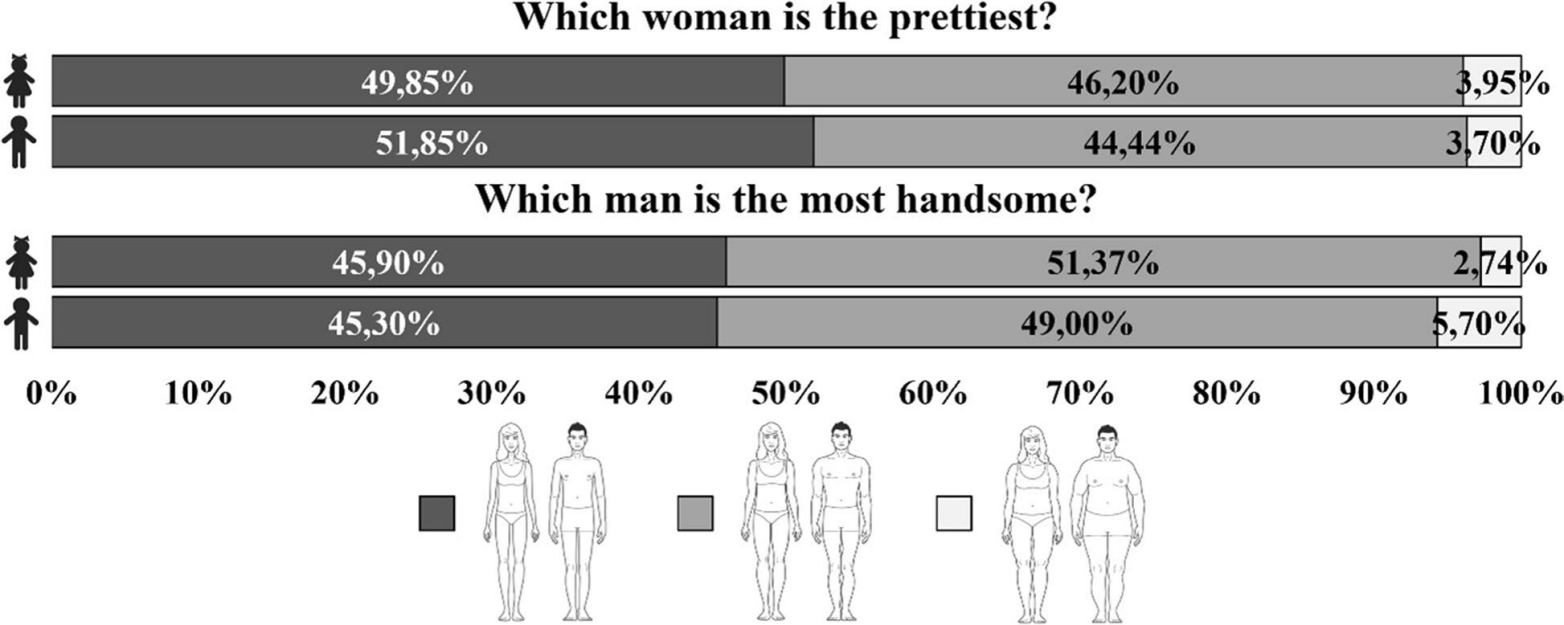
Assessment of the physical attractiveness of male and female silhouettes with different body types according to preschool girls and boys

First, we looked at how children assessed the overall physical attractiveness of individuals with different body types (questions: Which woman is the prettiest?/Which man is the most handsome?) (Fig. 1). For both female and male silhouettes, both girls and boys were least likely to indicate the obese bodies as most attractive. Slim bodies and those of normal weight were assessed as equally attractive. Compared with these two body types, the obese body type was selected the least frequently (*p* < 0.001).

### Ascribing attributes of happiness to different body types

**Fig. 2:**
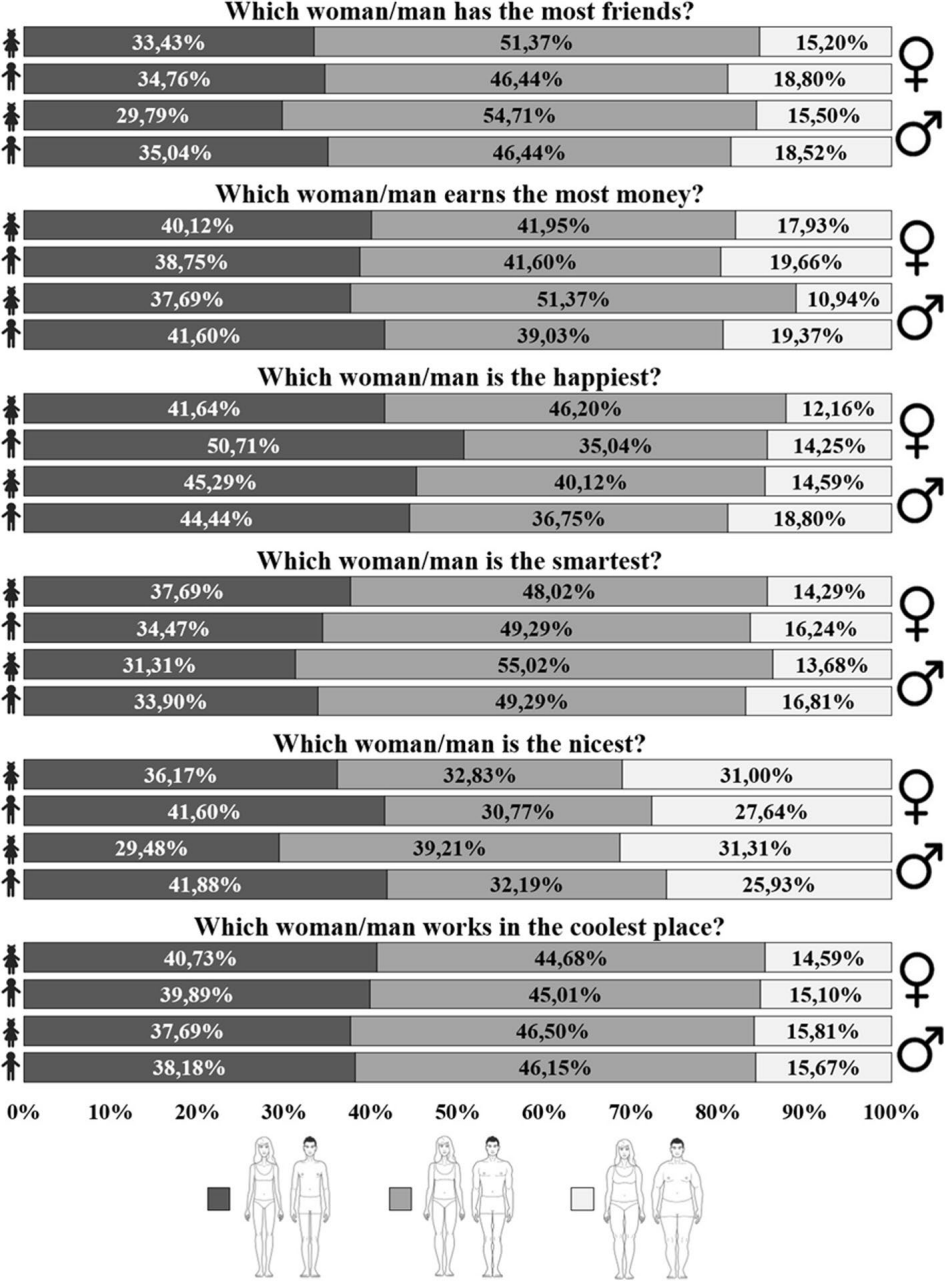
Assessment of specific attributes of happiness of the male and female silhouettes with different body types according to preschool girls and boys

First, we analysed the children’s assessments of happiness for each of the presented silhouettes (Fig. 2). Our results show that girls assessed slim and normal weight individuals similarly, while boys assessed the slimmest women as being the happiest (χ^2^ = 8.80, *p* = 0.012, *V* = 0.11). Next, we analysed the assessment of other specific happiness attributes for different silhouettes. Here, we observed gender effects with regard to the children’s choices of which type of man earned the most money. Girls also assessed normal weight men as likely to earn the most money and were more likely to give this assessment than boys (χ^2^ = 14.29, *p* =  < 0.001, *V* = 0.15). Differences were also observed regarding the choices concerning assessment of niceness of the silhouette in the case of girls, seven obese men were described as nice, whereas boys were more likely than girls to assess slim men as nice (χ^2^ = 11.35, *p* = 0.003, *V* = 0.13). The results clearly suggest that children associate looks with happiness, indicating that the body type assessed as the most physically attractive corresponded to the consistent selection of the same body type in questions regarding happiness.

In our study, we controlled for SES (the family’s area of residence and parental level of education) and familial factors (the number of siblings) and no statistically significant differences were observed.

**Fig. 3:**
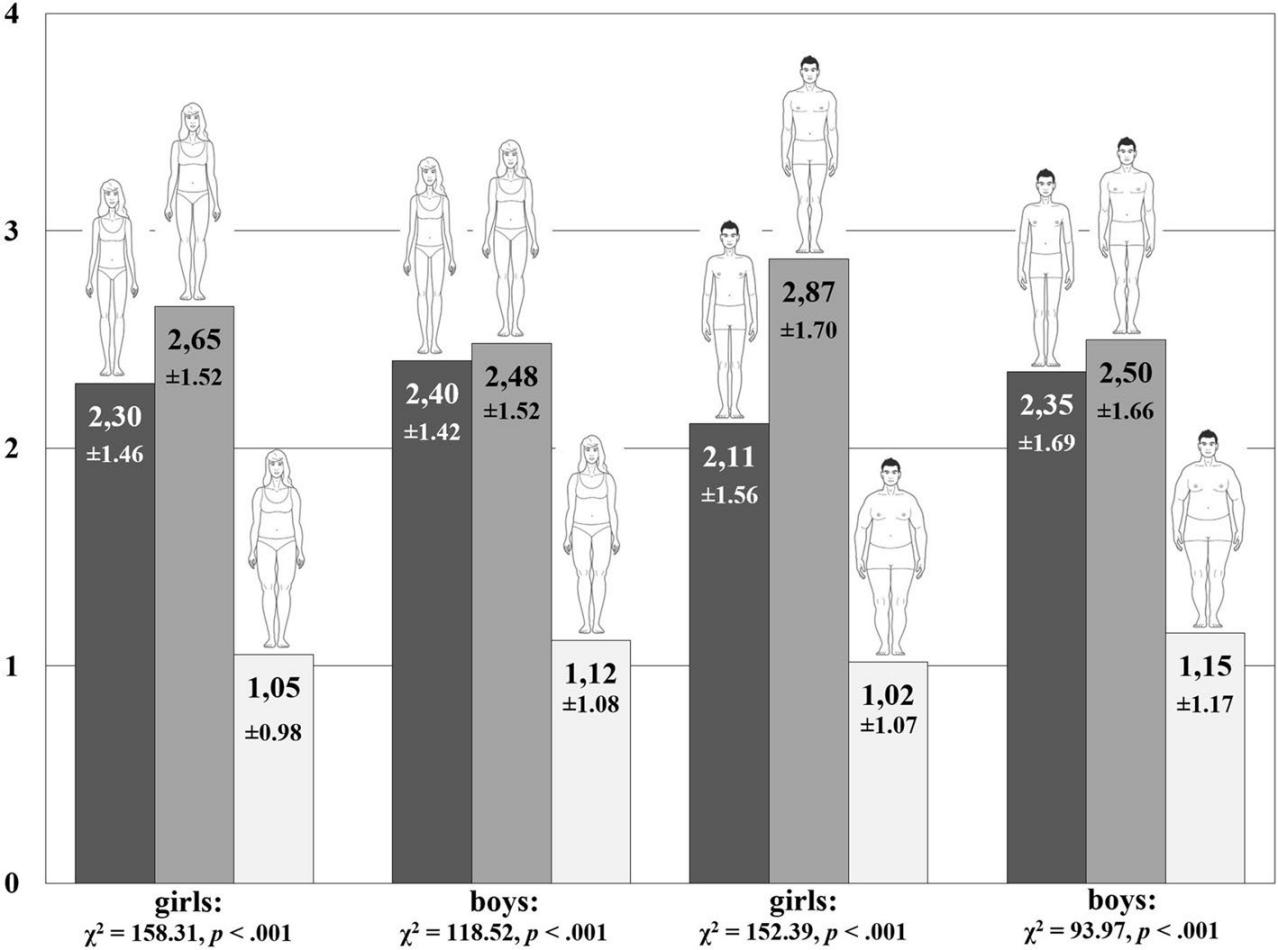
The attributes of happiness ascribed to different body types by children

In the next step, we analysed the overall happiness scores and their relationships with each body type according to preschool girls and boys. Our results suggest that children (regardless of their sex) generally strongly associated body type with happiness (Friedman’s ANOVA). Slim and normal body weight were similarly strongly associated with general happiness, especially among boys. Girls were more likely to ascribe happiness to normal-weight women (*t* = 2.27, *p* = 0.024) and normal-weight men (*t* = 4.45, *p* < 0.001). It can be seen (Fig. 3) that the lowest happiness scores were ascribed to obese body types, but girls assessed men with a normal body type as happier than boys (*t* = 2.87, *p* = 0.004).

### Ascribing attributes of happiness and their relationships with the physical attractiveness of different body types

The five-year-olds chose the same body types as the prettiest/most handsome and happiest (see Table 1).

**Table 1:** The association between selecting a male or female body type as the prettiest/most handsome and their selection with regard to questions about happiness

Questions	Which woman is the prettiest?						Which man is the most handsome?					
	girls			boys			girls			boys		
	χ^2^	*p*	V	χ^2^	*p*	V	χ^2^	*p*	V	χ^2^	*p*	V
Which woman/man has the most friends?	33.48	< .001	0.23	17.04	.002	0.16	57.09	< .001	0.29	41.74	< .001	0.24
Which woman/man earns the most money?	33.88	< .001	0.23	65.02	< .001	0.30	51.31	< .001	0.28	52.89	< .001	0.27
Which woman/man is the happiest?	115.31	< .001	0.42	77.04	< .001	0.33	67.59	< .001	0.32	117.32	< .001	0.41
Which woman/man is the smartest?	3.54	n.s	0.07	1.37	n.s	0.04	25.93	< .001	0.20	8.64	n.s	0.11
Which woman/man is the nicest?	22.58	< .001	0.19	13.43	.009	0.14	22.69	< .001	0.19	38.65	< .001	0.23
Which woman/man works in the coolest place?	54.34	< .001	0.29	60.24	< .001	0.29	34.76	< .001	0.23	51.63	< .001	0.27

The results clearly suggest that children associated looks with happiness, indicating a body type as the most physically attractive and also as the happiest. Detailed analysis of the answers to every question revealed that children did not associate one’s looks with happiness for the “smart” category. This was especially true for female bodies, as the assessments by both boys and girls were not statistically significant. Hence, the perception of the female body as attractive is not associated in any way with describing her as a smart or “wise” person. The only exception was the assessment of male bodies by girls —33.13% considered the normal weight body type to be the most attractive and associated it most strongly with wisdom, while 21.28% thought the same about the slim male body type. The strongest associations for prettiest/most handsome occurred for the question about general happiness. In the case of girls, both slim women and normal-weight women were most frequently assessed as the happiest (both 31.61%). When the body types were assessed by boys, the results were similar (35.90% and 25.07%, respectively). In the case of male bodies, girls assessed the slimmest (30.40%) and average-weight (30.09%) men as the happiest. The boys made similar assessments (32.48% and 29.34%, respectively).

## Discussion

Currently, the media creates the standards for perfect bodies: slim for women, muscular for men. These standards are then internalised by both sexes already in their childhood as aspirations determining broadly understood happiness [50]. Children with average or muscular builds are perceived as happy, kind, strong, neat, and popular. Overweight children, at least in the Western world, are assessed as being clumsy, lazy, stupid, and more likely to cheat [51]. In our study, we shed light on the relationship between associations of obesity and happiness made by preschool girls and boys (5-year-olds).

### Slim = Good

In our study, both boys and girls assessed obese bodies as unattractive. Hence, we found support for the first hypothesis (H1)—preschool children generally prefer slim and average-build bodies over obese bodies. Contrary to previous results Kościcka and Czepczor [52] the participants of our study did not find the emaciated silhouette unattractive -while overly obese bodies are broadly recognized as unattractive, slim bodies, regardless of the degree of slimness, are still associated with various attributes of happiness.

### Women must (not) be slim?

The second hypothesis (H2), expecting that five-year-olds would have specific requirements regarding the female body (in order to be happy, she needs to be slim), was partially confirmed. For the general question about happiness, both boys and girls identified slim women as happy; however, girls also considered the possibility of average-weight women being happy, which signalizes their healthier standards with regard to weight in women.. Interestingly, in this category, girls also assessed normal-weight men as being more predisposed towards happiness (and not slim men). These results are somehow in opposition to the works by Harriger and Calogero [53], who found that preschool children manifest reluctance towards both obese and average-build bodies, suggesting that the belief that ‘slimmer is better’ appears at increasingly younger ages [38]. In our study, we did not observe such a relationship. Moreover, when the girls were asked about which man was the nicest, they did not make distinctions based on body type. There were also no significant differences with regard to the assessments of women with different body types. It is worth noting that perceiving obese people as funny [30], making up for their weight with jokes and playfulness, is also a stereotypical perception of obese people [54]. There is clear evidence from the social science literature that fat stigmatization exists in all realms of life [29, 30, 55, 56] and that it mostly concerns women.

It could be that the ‘kind’ category and its definition in five-year-old girls is problematic, considering their stage of moral development. In the case of boys, there was a clear distinction: only slim men were assessed as kind.

The developmental pattern observed in our research is worth noting: when a child primed themselves by choosing a body type as the most attractive, they selected the same body type as the happiest.

### “Pretty but not necessarily smart” women

Our findings also show that boys did not associate looks with happiness in the ‘smart’ category when assessing female silhouettes. There were no significant differences between assessments of seeing different female body types as wise. Seeing a woman as pretty was in no way associated with perceiving her as a ‘wise’ person. The only exception was the assessment of male bodies by girls: one-third of the girls assessed the normal weight body type as being the wisest and most attractive, and one-fifth selected the slim body type. It can thus be concluded that the “if she’s pretty, then she’s less smart” stereotype is already present in children at the age of five years. Other researchers have reported similar results [57, 58]. Interestingly, the average male body type, followed by the slim body type, was assessed by girls as the wisest. This in turn already indicates that wisdom as a category is more linked with male look that with female look.

## Limitations

Our study used illustrated scales to study the assessment of body types. This is in line with the generally used methodology because pictorial and/or photographic scales are the most commonly used scales when performing studies with preschoolers. Many authors claim that photographic scales are advantageous when studying preschoolers’ attitudes towards body types because they foster children’s immersion in the task and identification with the picture [59]. Meers and Koball [59] showed that children express more prejudice towards drawn silhouettes than silhouettes in photographs; however, this could be due to other factors associated with photographs. The authors suggested that cartoon silhouettes are associated with more stereotypical answers by children because the realism of such pictures is reduced.

The use of silhouettes in this study also led to other limitations. This method might not necessarily reflect the participants’ perceptions, since the technique obliges them to suppress their latent responses and select a solution from the scale. Additionally, Dunphy-Lelii and Hooley [60] indicated that if the same scale is used to assess a body type just after presenting an ideal body type, the latter influences the former. Children may also have difficulty making comparisons for a hypothetical ideal.

On the one hand, recruiting children of the same age (5 years old) was an advantage of this work; on the other hand, it may make it difficult to generalize the results for the population of preschool children.

## Conclusions

Our study shows that female bodies are already perceived along gender stereotypical lines at the age of 5 years, and are related to potential predictions concerning one’s happiness: if a woman’s body was assessed as beautiful, it was not associated with describing her as ‘wise’ (this effect was also present in men). Finally, the children assessed individuals with slim bodies, as well as those of normal weight, as happier than obese individuals. However girls also considered the possibility of average-weight women being happy, which signalizes their healthier weight standards.

The results of this study may have important practical implications for the quality of life of children and their families, as they may translate into changes in the eating habits of families, the development of children’s eating habits, etc., and in doing so, may influence their self-esteem, their own body, and the perceptions about the attractiveness of their body. Furthermore, the results may be important for the quality of the social relationships that children will form during the subsequent stages of their development. These can be influenced by their preferences with regard to individuals of a given gender and body type as playmates, someone to spend their free time with, romantic partners, etc. Thus, this research is in line with studies indicating the importance of the immediate environment (see [51, 61]) in the development of children’s perceptions of happiness and in their development of accurate patterns of perceptions of other people that are free from prejudice. Moreover, differences in the perception of the relationship between happiness and the appearance of men and women indicate the need to implement or continue gender awareness programs among preschool children.

## Data Availability

The datasets used and/or analysed during the current study are available from the corresponding author upon reasonable request.

## Abbreviations

SWB: Subjective well-being
B&H: Beauty & Health scale
